# Spontaneous brachial haematoma in a patient with ST elevation myocardial infarct: a case report of a rare side effect of thrombolysis

**DOI:** 10.1093/ehjcr/ytaf001

**Published:** 2025-01-15

**Authors:** Vasileios Bouratzis, Christos S Katsouras, Christos Floros, Ilektra Stamou, Lampros K Michalis

**Affiliations:** Second Department of Cardiology, University of Ioannina Medical School, University Campus, Stavros Niarchos Avenue, Ioannina 45 500, Greece; Second Department of Cardiology, University of Ioannina Medical School, University Campus, Stavros Niarchos Avenue, Ioannina 45 500, Greece; Second Department of Cardiology, University of Ioannina Medical School, University Campus, Stavros Niarchos Avenue, Ioannina 45 500, Greece; Second Department of Cardiology, University of Ioannina Medical School, University Campus, Stavros Niarchos Avenue, Ioannina 45 500, Greece; Second Department of Cardiology, University of Ioannina Medical School, University Campus, Stavros Niarchos Avenue, Ioannina 45 500, Greece

## Case presentation

**Figure 1:**
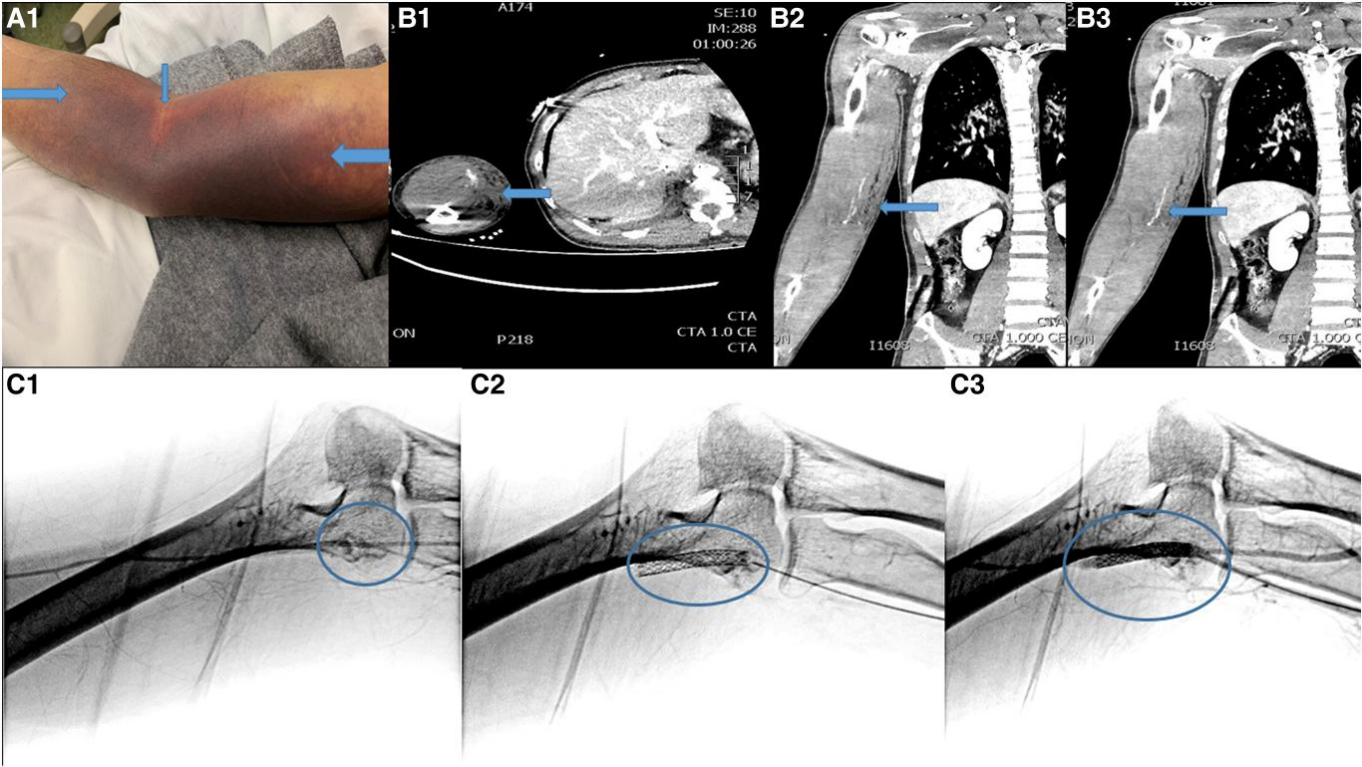
*Panel A1*: spontaneous haematoma of right brachial area, *panel B1*: transverse section of computed tomography angiography of right brachial artery showing extravasation and haematoma in right upper limb. *Panels B2-B3*: coronal section of Computed tomography angiography of right brachial artery showing extravasation and haematoma in right upper limb *Panel C1*: angiography of right brachial artery showing extravasation in right brachial artery, *Panel C2*: stent of right brachial artery. *Panel C3*: Angiography of the right brachial artery after deploying the stent, showing no extravasation. (arrows show the haematoma on the right upper limb in brachial area).

A 44-year-old male presented to a hospital without haemodynamic support facilities due to anterior ST elevation myocardial infarct and cardiogenic shock. He received intravenous thrombolysis with alteplase (15 mg bolus, 0.75 mg/kg over 30 min, and then 0.5 mg/kg over the next 60 min), along with orally 300 mg of clopidogrel and 250 mg of aspirin and subcutaneously 6000IU of enoxaparin. He was immediately transferred to the catheterization laboratory of our hospital. An intra-aortic balloon pump (Arrow, Morrisville, USA) was initially inserted through the left femoral artery. The right transfemoral coronary angiogram revealed three-vessel coronary artery disease. The culprit lesion (proximal left anterior descending artery) was treated with a drug-eluting stent. Eighteen hours after thrombolysis (12 h post coronary angioplasty), a large compartment haematoma was observed in his right arm (no previous attempt was made to insert an arterial line into the brachial artery for monitoring) (*Figure 1*: Panel A1). Haemoglobin levels decreased from 15 g/dL to 9 g/dL. Emergent computed tomography angiography (CTA) revealed contrast extravasation from the distal part of the brachial artery, accompanied by a haematoma (*Figure 1*: Panels B1-B3). A 5 Fr sheath was inserted in the right radial artery (via surgical exposure and micro-puncture set). A balloon expandable stent graft 5 × 38 mm (Bentley InnoMed GmbH, Germany) was deployed in the right brachial artery (*Figure 1*: Panels C1-C3). The patient was discharged after 10 days.

Haematomas in non-puncture sites are a rare complication of thrombolysis. Bleedings after thrombolysis can extend well beyond the expected timeframe based on plasma clearance of alteplase (initial half time < 5 min). The causes for this ‘delayed’ bleeding are not clear, but systemic coagulopathy is the most possible cause. Alteplase administration causes hypoplasminogenaemia, lower factor V, and sustained hypofibrinogenaemia (lasting at least 24 h).^1,2^ Depending on the related area of bleeding and haemodynamic status, treatment can be conservative, and involve haemostatic surgery, embolization or angioplasty of the responsible vessel.^3^

## Data Availability

The data underlying this article are available in the article and its online supplementary material.

## Notes

**Consent:** The authors confirm that written consent for submission and publication of this case report including images and associated text has been obtained from the patient in line with Committee on Publication Ethics guidance.

**Funding:** None declared.

## References

[1] Huang X, Moreton FC, Kalladka D, Cheripelli BK, MacIsaac R, Tait RC, et al Coagulation and fibrinolytic activity of tenecteplase and alteplase in acute ischemic stroke. Stroke 2015;46:3543–3546. 26514192 10.1161/STROKEAHA.115.011290

[2] Vandelli L, Marietta M, Gambini M, Cavazzuti M, Trenti T, Cenci MA, et al Fibrinogen decrease after intravenous thrombolysis in ischemic stroke patients is a risk factor for intracerebral hemorrhage. J Stroke Cerebrovasc Dis 2015;24:394–400. 25497721 10.1016/j.jstrokecerebrovasdis.2014.09.005

[3] Rossaint R, Afshari A, Bouillon B, Cerny V, Cimpoesu D, Curry N, et al The European guideline on management of major bleeding and coagulopathy following trauma: sixth edition. Crit Care 2023;27:80. 36859355 10.1186/s13054-023-04327-7 PMC9977110

